# Supplementary material for: Are carbon emissions trading and green financial instruments synergistic? -Comprehensive quantitative research based on content analysis
Source: PLoS One. 2024 Mar 7;19(3):e0298601. doi: 10.1371/journal.pone.0298601 (PMC10919601; doi:10.1371/journal.pone.0298601)
Supplement: S1 Data — (ZIP) [file pone.0298601.s001.zip › Data/地级市Z_F工作报告.zip!/╔╜╢1⁄2/═■║ú╩╨/└┤╫╘╣1⁄2╓┌║┼í╛┬φ┐╦╩2╛▌═°í┐_2013.docx]

各位代表：

现在，我代表威海市人民政府向大会报告工作，请予审议，并请各位政协委员和其他列席人员提出意见。

一、2012年政府工作回顾

过去的一年，在省委、省政府和市委的坚强领导下，市政府和全市人民一道，以邓小平理论、“三个代表”重要思想、科学发展观为指导，深入贯彻落实党的十八大和省第十次党代会、市第十四次党代会精神，紧紧围绕现代化幸福威海建设，坚持生态立市、科教兴市、产业强市，全力做好稳增长、控物价、调结构、惠民生、抓改革、促和谐的各项工作，经济社会发展呈现稳中有进的良好态势。

（一）促进经济健康发展，转型升级步伐进一步加快。面对严峻复杂的经济形势，牢牢把握稳中求进的工作总基调，千方百计化解发展难题，促进经济平稳运行。预计全市生产总值增长9.5%左右，公共财政预算收入增长16.1%，城镇居民人均可支配收入增长13.2%，农民人均纯收入增长13.2%，居民消费价格涨幅控制在1.8%左右，三次产业比重由8.1：54：37.9调整为8.2：53.6：38.2。强化科技兴农，建立新品种、新技术示范基地181处，推广测土配方施肥236.5万亩，粮食产量稳定在100万吨以上，渔业经济总产值预计增长8%。突出工业带动，促成38个产业合作项目，完成技术改造投资440亿元，增长16%；预计高新技术产业产值增长18.8%，占工业总产值的比重达到35.5%，提高1.2个百分点；规模以上工业增加值增长10.5%。推动服务业上档升级，积极创建全国旅游标准化示范城市，成功举办国际食品博览会、国际渔具博览会、海峡两岸食品博览会、中日韩文化创意产业博览洽谈会，大力推进中心商圈建设，社会消费品零售总额增长15%，旅游总收入增长17.5%，港口吞吐量增长12.7%。大力开拓国际市场，外贸进出口总额达到171.2亿美元，其中出口106.6亿美元。深入开展自主创新年活动，促成产学研合作项目133项，新认定各类创新型企业58家，引进各类人才1.8万人，成为国家知识产权质押融资试点城市。

（二）扎实办好民生实事，社会保障水平进一步提高。全市财政民生支出增长22.7%，占财政总支出的比重达到60.5%，新增财力主要投向了民生领域。完善就业扶持政策和困难群体就业援助制度，扶持创业1145人，带动就业4000人，被评为全国首批创业先进城市。提高新农合和城镇居民医保的财政补助标准，城镇职工医保年度最高支付限额由40万元提高到50万元，医疗卫生保障服务水平进一步提升。健全城市“三无”人员政府供养保障机制和高龄津贴制度，提高城乡低保标准、困难群体医疗救助标准，困难群众生活得到有效保障。实施棚户区改造1.2万户，建设保障性住房4829套。创新校车运营模式，增加新模式校车186辆，新建10所公办性质幼儿园，全面完成校舍安全工程，建成5个职业教育示范实训基地，实施教师素质提高工程，教育基础保障能力进一步增强。积极为群众性文化活动的开展创造条件，新开放4个文化艺术中心，新建文化大院106个。优先发展公共交通，购置节能环保型公交车79辆，开通公交旅游观光专线。完成供热计量和建筑节能改造786.4万平方米，改善6.4万户居民的供暖条件。中心城区拆除违法建筑170处，规范清理户外广告，优化了城市综合环境。完成365个村的环境综合整治，新建和改造农村住房1.6万户，实施农村自来水工程38处，改善了农村生活条件。

（三）大力推进招商引资，项目建设力度进一步加大。强化招商引资目标责任制，不断优化投资环境，推动招商引资工作取得显著成效。全年实际到账外资8亿美元，规模再创新高，外资结构明显优化。实际利用内资481.6亿元，增长15%。百项市级重点项目完成投资330亿元，威高骨科材料、豪雅光电、日月光半导体三期等33个重点产业项目建成投产。青荣城际铁路、威海新港、江家寨立交桥、统一路南延等基础设施项目扎实推进。金线顶区域整体开发、宝泉广场建设、环翠楼公园改造等38项城建重点工程加快实施。预计全市固定资产投资达到1595亿元，增长20.2%。

（四）统筹区域协调发展，重点领域改革进一步深化。以创新的思路和举措优化空间布局，调整经济区划，实施东部滨海新城、双岛湾科技城、威海南海新区、好运角旅游度假区、乳山滨海新区、里口山风景名胜区等六大重点区域开发建设，拉开市域一体化发展大框架。设立市级蓝色经济区建设专项资金，争取37个项目列入省重点扶持项目，蓝色经济区建设9个重大事项取得实质性进展。启动10个示范镇培育计划，其中4个镇列入全省“百镇建设示范行动”示范镇。理顺城市建设管理体制，建成城市综合管理信息系统，在中心城区99平方公里范围内推行网格化、数字化的城市精细化管理模式。组建城市开发投资公司和水利建设投资公司，加强国有资产运营管理。推动金融业态创新，成立融资仓储和融资租赁公司，开展威海农村信用体系试验区建设，帮助企业破解融资瓶颈。医药卫生体制改革、文化管理体制改革、事业单位改革及农村领域、社会领域的改革扎实推进。

（五）加强生态文明建设，资源环境保护进一步强化。健全生态环境规划，出台规范土地利用、海域使用管理办法，强化资源集约节约利用。实施威海湾养殖清理、黄垒河综合整治、海域海岸整治、河道生态治理工程，优化了水生态环境。继续整治建筑垃圾、工地扬尘、机动车尾气等问题，市区空气质量优良率达到96.4%。治理规模化畜禽养殖场372家。实施道路绿化工程，成片造林8.1万亩。预计万元生产总值能耗降低3.7%，主要污染物排放量继续下降。顺利通过国家环保模范城市群复核验收。

（六）加快推进文化建设，各项社会事业进一步加强。坚持全民参与、共建共享，积极创建全国文明城市，年度城市文明指数位居69个新参评地级城市首位。开展幸福威海文化活动，扎实推进“十艺节”筹备工作。成功承办长距离铁人三项世界杯赛和亚太霍比帆船锦标赛。深入推进“平安威海”建设，安全生产、食品药品安全监管、应急管理、社会管理等工作得到加强，社会保持和谐稳定。国防动员、人民防空、民兵预备役工作进一步加强，人口计生、民族宗教、妇女儿童、残疾人、外事、侨务、对台、档案、史志、地震、气象、仲裁、慈善等事业也取得新成绩。

（七）加强政府自身建设，行政服务环境进一步优化。扎实开展机关作风建设年活动，加快建设法治政府、服务型政府、廉洁政府。修订政府工作规则，出台重大行政决策程序规定和规范性文件管理办法，推行行政复议委员会试点工作，政务运行更加规范有序。开展“千家企业大走访”活动，为企业解决实际问题1380个；精简行政许可事项，减免行政事业性收费23项，减轻企业负担1.6亿元。自觉接受人大的法律监督、政协的民主监督和群众的社会监督，办理人大代表建议和政协委员提案238件，办复率达到100%。扎实推进科技防腐，廉政建设不断深入。

各位代表！新一届政府的起步年能够实现良好开局，得益于全市人民的共同努力和社会各界的大力支持。在此，我代表市人民政府，向全市人民，向人大代表和政协委员，向各民主党派、工商联、人民团体和无党派人士，向驻威人民解放军、武警官兵，向各位老领导、老同志，向所有关心支持威海发展的海内外朋友，致以崇高的敬意和衷心的感谢！

在总结成绩的同时，我们也清醒地看到，我市经济社会发展中仍存在一些矛盾和问题，主要表现在：制造业和服务业的发展层次有待提升，企业做大做强的能力不强，经济稳定增长的基础还不够稳固；稳定外需的难度较大，扩大内需的动力不足；高新技术企业数量不多，有自主创新能力的企业比较少；产业结构、财税结构都有待优化，经济运行质量和效益仍需提高；资源节约和环境保护工作仍需加强，完成节能减排约束性指标的压力比较大；城乡区域发展还不够平衡，城镇化质量仍需进一步提高；一些关系群众切身利益的问题有待解决；少数政府工作人员推诿扯皮、效率低下，行政服务环境有待优化；消极腐败现象在一些领域仍然存在，廉政建设有待加强。对此，我们一定认真对待，不回避矛盾，不推卸责任，采取逆向切入的方法，从解决问题入手推动各项工作，让政府工作更加扎实、更富实效。

二、2013年政府工作的目标要求

各位代表！今年是全面贯彻落实十八大精神的开局之年，是实施“十二五”规划承前启后的重要一年。综合判断，国内外经济形势依然错综复杂、充满变数，经济发展面临的困难仍然较多，各种挑战不能低估，但经济社会发展仍具备很多有利条件和积极因素。只要我们坚定信心，把握有利机遇，沉着应对挑战，就一定能够攻坚破难，不断开创科学发展的新局面。

按照市第十四次党代会和市委十四届二次、三次全体会议精神，2013年政府工作的总体要求是：深入学习和全面贯彻落实党的十八大精神，以邓小平理论、“三个代表”重要思想、科学发展观为指导，紧紧围绕主题主线，以提高经济增长质量和效益为中心，坚持生态立市、科教兴市、产业强市，进一步深化改革开放，强化创新驱动，优化经济结构，更加注重保障和改善民生，培育形成新活力、新动力、新体系、新优势，推动市域一体化发展，实现经济持续健康发展和社会和谐稳定，加快建设现代化幸福威海。

主要预期目标是：生产总值增长10%，公共财政预算收入增长13%，固定资产投资增长20%，社会消费品零售总额增长15%，外贸进出口增长5%，实际利用外资稳定增加，实际利用内资增长15%，城镇登记失业率控制在2%以内，城镇居民人均可支配收入和农民人均纯收入均增长11%，居民消费价格涨幅控制在3.5%左右，人口自然增长率控制在1‰以内，万元生产总值能耗下降3.7%，化学需氧量、氨氮、二氧化硫、氮氧化物排放量分别下降0.5%、2%、3.5%、5.5%。

上述预期目标的确定，充分考虑到经济形势的复杂性、严峻性和多变性，考虑到我市的发展基础、现实条件和实际需要，力求积极稳妥，切实可行。在实际工作中，我们将继续把握好稳中求进的工作总基调，落实“有条件的地方在现代化建设中继续走在前列”的要求，尽可能加快发展步伐，追求尊重经济规律、有质量、有效益、可持续的速度；全面落实五位一体总体布局，更加自觉、更加坚定地推进改革开放，加快实施蓝色经济区建设、六大重点区域开发等重大战略，促进工业化、信息化、城镇化、农业现代化同步发展；坚持把实干精神贯穿于政府工作始终，健全有利于科学发展的目标体系、考核办法和奖惩机制，转变作风，真抓实干，使发展成果更多、更公平惠及人民群众，使政府工作更加符合科学发展的要求，更加符合人民群众的期待。

三、促进经济持续健康发展

坚持产业强市，着力激发各类市场主体发展新活力，增强创新驱动发展新动力，构建现代产业发展新体系，培育开放型经济发展新优势，促进经济增长速度与质量效益相统一。

（一）突出工业带动。坚持走新型工业化道路，推动信息化和工业化深度融合，传统产业改造与新兴产业培育双轮驱动，稳定工业经济增长。实施工业转型跨越工程，改造提升机械制造、电子信息、纺织服装、食品、橡胶制品等传统优势产业，巩固工业经济基础；加快发展战略性新兴产业，提升工业结构层次；培植20家领军企业、50家骨干企业、30家高成长性企业，壮大优势企业群体；提高医疗器械、数控机床、智能终端及打印设备、船舶、汽车及零配件、海洋食品、轮胎、地毯、家纺、渔具等10大类优势产品的竞争力，集聚关联产品，增创规模优势；打造军民结合产业基地，拓宽企业市场空间。实施制造业信息化科技工程，培育10家“两化”融合示范企业。支持工业企业技术改造，重点实施威高集团高端医疗用品、海鑫新材料、新北洋专用打印机等45个高新技术项目，抓好三角集团高性能全钢载重子午胎、中航威海船厂扩建、蓝岛科技新材料、海之宝海藻食品深加工等58个升级改造项目，确保当年开工一批、竣工一批，以有效投入夯实工业经济基础。工业增加值力争增长11%。

（二）发展现代农业。落实强农惠农富农政策，促进农民持续增收。创新农业经营体制机制，鼓励发展多种形式的新型农业经营主体，扶持农业龙头企业，培育30个市级农民专业合作示范社，提高农业组织化和规模化程度。加快国家现代农业示范区建设，继续开展农业科技结对活动，实施农业科技成果转化与良种工程，建成土壤肥料院士专家工作站，增强农业综合生产能力。落实“菜篮子”负责制，推进27个粮油高产创建示范片建设，稳定粮食产量。建设20个现代渔业特色园区，新增专业性远洋渔船80艘以上。加强绿色生产，从源头上确保农产品质量安全。

（三）壮大现代服务业。把加快服务业发展作为优化产业结构的突破口，推进服务业标准体系建设，支持企业剥离非核心业务，大力培育新型业态，促进服务业增量扩容。深化中韩陆海联运业务，支持发展现代物流业和临港产业，港口吞吐量增长13%。培育现代信息技术产业，推动新北洋等5个省级重点服务外包企业加快发展，支持物联网、软件产品开发应用。培育融资仓储、融资租赁等新型金融业态，加快多层次金融体系建设，金融机构存贷款增长10%以上。发展楼宇经济，打造特色商圈，提升商贸服务业发展层次。大力发展文化产业，培育新型文化业态，鼓励创意产业发展。推进雨润北海湾等6个旅游重点项目，完善旅游公共服务设施，扩大旅游整体形象宣传，创建全国旅游标准化示范城市，旅游总收入增长13%。加快推进环翠区省级服务业综合试点工作。服务业增加值占生产总值的比重确保提高1个百分点，力争提高2个百分点。

（四）实施创新驱动战略。落实各级鼓励科技创新的政策，完善以企业为主体、市场为导向、产学研结合的技术创新体系，引导企业加大研发投入，增强核心竞争力，争取全社会研发投入占生产总值的比重提高0.1个百分点。更加注重协同创新，深入推进产学研合作。促进科技与金融紧密结合，稳妥开展国家知识产权质押融资试点工作。加强知识产权保护，出台科技成果转化和专利扶持政策，启动知识产权优势企业培育工程，发明专利授权量增长15%。推进产学研创新示范工程和“特聘专家”资助计划，引进一批高层次人才、专家团队。省级以上研发平台增加11家，高新技术企业力争新增20家以上，高新技术产业产值比重提高1个百分点。

（五）强化招商引资。把招商引资作为增强发展后劲的有效途径，坚持内外并重、上下联动，落实招商引资目标责任制，强化各市区和经济园区的招商主体责任，进一步加大招商引资力度。创新招商方式，实行小分队跟踪、专业化促进，瞄准重点区域、重点产业、重点企业开展合作。实施差异化招商战略，积极推进定向招商。强化以商招商，鼓励龙头企业引进配套企业、关联企业，推动企业增资扩股。支持企业境外上市，加强与境外股权基金、投资公司、私募基金的合作，促进利用外资方式多样化。推动引资、引技、引智有机结合，提高利用外资整体效益。

（六）加快项目建设。把项目建设作为扩大内需的重要举措，发挥好投资对经济增长的关键作用。今年组织实施100项市级重点项目，年内计划投资389亿元；实施103项重点工业投资项目，年内计划投资180亿元；中心城区实施六大系列36项城建重点工程，年内计划投资295亿元。在谋划项目建设中，既通过政策扶持引导民间投资，鼓励各类市场主体扩大投资，又注重打基础、利长远、惠民生，适度加大公共投资力度，使投资规模进一步扩大，项目结构进一步优化。落实项目推进责任制，强化对项目签约、开工、投产、膨胀、储备各环节的联动服务，以项目高效实施促进经济发展和民生保障。

（七）扩大消费需求。落实各项鼓励消费政策，适应居民消费结构升级的需要，积极发展社会养老、家政服务、体育健身、文化娱乐等消费性服务业，加快培育网络购物等新型消费业态，增强消费对经济增长的基础作用。完善流通网络，不断提高本地农产品的超市化、品牌化经营程度。实施“百展市场开拓计划”，办好国际食品博览会、国际渔具博览会等展会，帮助企业开拓国际国内市场。加强城乡消费设施和服务体系建设，加快推进宝泉广场、九龙城休闲购物广场等商贸项目，新增大型商业设施面积40万平方米。强化市场监管，规范市场价格和收费秩序，进一步优化消费环境。

（八）提高开放型经济水平。实施更加积极主动的开放战略，加快转变对外经济发展方式。优化出口结构，促进加工贸易转型升级，争创国家纺织品、渔具出口基地，加快形成以技术、品牌、质量、服务为核心的出口竞争新优势。培育进出口一体化企业，增加先进设备、能源资源、适销消费品进口比重。支持优势企业安全高效“走出去”，购买国际知名品牌和先进技术，设立研发中心、设计中心，推进境外资源开发和工程承包，提高企业国际化经营能力。积极推进出口加工区转型升级，打造开放型经济发展的新载体。

（九）激发企业发展活力。坚持把培育市场主体作为增强经济发展活力的根本基础，按照“抓大不放小”的思路，支持企业发展，鼓励全民创业。研究出台扶持政策，助推骨干企业做大做强，新增60家规模以上工业企业。开展“品牌提升年”活动，支持企业实施质量和品牌战略，争创省级以上名牌产品和驰名商标22个。扩大科技支行试点，助推科技型中小企业成长。实施中小微型企业“百千万”梯次成长计划，启用市中小企业公共服务中心，壮大中小企业群体。落实小额担保贷款贴息、税费减免等创业扶持政策，建设2处大学生创业孵化基地，不断催生新的市场主体。

四、统筹区域协调发展

坚持市域一体化发展，以蓝色经济区建设为引擎，深入实施重点区域带动战略，加快新型城镇化进程，提升区域资源配置和要素集聚功能，统筹城乡协调发展、区域联动发展。

（一）加快重点区域开发建设。六大重点区域是市域一体化发展的重要节点，是优化城市空间布局的战略选择，也是县域经济跨越发展的重要载体。坚持“规划引领、产业强区、创新驱动、生态优先、联动发展”，加快打造区域发展的新板块、现代产业的隆起带。按照一次规划、分步实施的思路，对重点区域实施统一规划，突出战略重点，统筹利用空间资源，增强规划的科学性和前瞻性。强化生态、生产、生活“三生”共融的理念，科学规划功能分区，搞好土地储备，预留发展空间，推动有序开发。坚持基础设施先行，统筹推进各类公共设施建设。鼓励先行先试，扩大各区域的管理权限，探索建立市场化管理与服务的新机制。研究出台专项扶持政策，引导各类资源向重点区域聚集。推动重点区域间加强合作，实现优势互补、产业互动、资源共享。

（二）深入推进蓝色经济区建设。强化园区、基地和企业的载体作用，着力构建现代海洋产业体系，培育海洋经济发展新优势。大力培植海洋新兴产业，支持发展海洋药物、海洋保健品和海洋生物新材料等高新技术产业。支持船舶工业调整产品结构，增强企业技术创新能力。推动海洋生物产业园建设，打造特色海洋经济园区。充分发挥蓝色经济区建设的集聚效应，用好专项资金和扶持政策，支持蓝色经济重点项目建设。加快推进国家海产品质检中心、山东船舶技术研究院、海洋商品国际交易中心等14个重大项目建设，不断增强海洋经济发展的保障能力。

（三）加快推进城镇化建设。坚持以提质加速、城乡一体为目标，以人的城镇化为核心，着力提高城镇化质量。实施市域规划编制工程，完善城镇化发展规划。突出内涵提升，促进中心崛起，不断提高中心城市的建设管理品质和辐射带动能力。突出产业支撑，落实以市为单位整体提升县域经济发展水平的要求，促进荣成、文登、乳山城市建设和产业结构优化升级，提高次中心城市的竞争力。突出示范带动，全面推进示范镇和中心镇建设，增强小城镇产业发展、公共服务、吸纳就业、人口集聚功能。促进园区规范有序发展，增强城镇集聚功能，拓宽城镇发展空间。深化户籍、土地管理制度改革，有序推进农业转移人口市民化。

（四）扎实推进新农村建设。坚持把解决“三农”问题作为重中之重，强化以城带乡，促进基本公共服务均等化，推动城镇基础设施向农村延伸，产业向农村拓展，服务向农村覆盖。实施农村公路网化工程，改造2046公里乡村路。实施新一轮农网改造工程，新建电气化村85个。加快实施小型农田水利重点县项目，完成50座病险塘坝除险加固。实施农村饮水安全工程，解决14.5万人口的饮水安全问题。

（五）加强基础设施建设。加快青荣城际铁路工程进度，推进城市内环路、城市综合交通枢纽、威海港疏港公路、文莱高速公路、威石辅路等项目，江家寨立交桥竣工通车；规划论证国际航运服务中心建设，推进港口资源整合，抓好重点港口建设，威海港国际客运中心投入使用；开工建设16个电网项目；实施9个燃气工程和10个热力工程；推进泊于水库、南水北调续建配套工程等水利项目；中心城区加快推进金线顶、五渚河、九龙湾等区域整体开发，启动市民中心、海源公园建设，新建改造37条城市道路，进一步增强基础设施保障能力。

五、强化生态文明建设

坚持生态立市，牢固树立尊重自然、顺应自然、保护自然的生态文明理念，加强生态文明制度建设，加大资源环境保护力度，争创国家生态市，促进生产空间集约高效、生活空间宜居适度、生态空间山清水秀，努力成为“美丽中国”的示范区。

（一）加强自然资源保护利用。落实最严格的耕地保护制度，推进城乡建设用地增减挂钩和工矿废弃地复垦调整利用，有序拓展用地空间。严格执行建设用地投资强度标准，加大用地用海批后监管力度，提高集约利用水平。出台海域使用权转让出租管理办法，培育、规范海域资源市场。推行海岸带网格化管理，实施海域海岛海岸带修复工程，严厉查处盗挖海砂、非法养殖、排污倒废等违法行为。

（二）大力推进节能减排。严格落实节能减排目标责任制，按照控增量减存量的思路，控制能源消耗强度，削减污染物排放总量。严守产业红线，从源头上控制“两高一资”项目，加快淘汰落后产能。强化重点用能企业能源审计和清洁生产审核，提高工业固体废物、餐厨废弃物综合利用水平。加强对重点排污企业的监管，引导企业落实环境保护主体责任。推进污水处理厂建设和升级改造。建立区域大气污染联防联控机制，加强重点燃煤企业脱硫脱硝设施改造和运行管理，加快淘汰“黄标车”。

（三）持续提高生态环境质量。扎实开展保障群众健康环保专项行动，加大对建筑施工噪声、工地扬尘、散流物体运输、危险废物等重点领域的整治力度，切实解决损害群众健康的环境问题。加强城乡水源地保护，实施237条农村河道生态化治理，推进现代水利示范市、水生态文明城市建设。深入开展农村环境保护综合整治，控制农业面源污染。加强水土保持和湿地生态保护，推进3个山体治理修复项目。实施水系、道路、村庄等绿化工程，提高森林防火和病虫害防控能力，成片造林7万亩。完善海洋功能区规划，提高海洋防灾减灾能力，争创首批国家海洋生态文明示范区。

六、扎实推进重点领域改革

坚持改革创新，更加尊重市场规律，更好发挥政府作用，坚定不移、不失时机地深化各项重点领域改革，最大程度激发体制机制活力，不断增强发展的内生动力。

（一）健全公共资源管理机制。按照集中交易、管办分离的原则，整合各类公共资源交易市场，建立统一规范的公共资源交易中心，加强监督管理，实现规范运行。深化资源性产品价格改革，健全资源有偿使用制度。发挥国有投资公司的作用，强化国有资产管理和运营。加强经营性国有资产监管，规范机关事业单位合同管理，确保国有资产保值增值。

（二）推进财税管理体制改革。落实结构性减税措施，大力加强财源建设。依法规范收入管理，创新税源管控机制，加强非税收入管理，努力提高“四税”比重，促进财税收入总量质量双提高。继续推进预算制度改革，健全财政支出绩效评价和投资评审机制，规范公务支出行为，提高财政资金使用效益。

（三）创新金融管理服务方式。引导和鼓励信贷资金投向实体经济，加大对中小微型企业和“三农”的服务力度，切实降低企业融资成本。创新企业抵质押担保方式，开展保险基层服务体系建设试点，提升金融服务效率。推动债券融资和三角轮胎等重点企业上市，扩大直接融资规模。规范民间融资，开展民间融资服务公司试点工作。

（四）完善城市管理体制。拓展城市综合管理信息系统的应用范围，建立城市管理督导巡查制度，加强城市精细化管理。积极推进“智慧城市”建设，规划建设公共服务云平台，启动智能交通管理系统建设，提升城市智能化、信息化水平。扎实推进城乡社区建设，创新社区管理机制，推广“12349”社区居家服务模式，增强社区服务功能，方便群众生活。

（五）加强和创新社会管理。深化“平安威海”建设，完善立体化社会治安防控体系，强化司法基本保障，依法防范和惩治违法犯罪活动。大力推行安全生产网格化管理，加强渔船作业、道路交通等领域专项整治，遏制重特大安全事故。健全应急管理体系，增强突发事件应急处置和救援能力。不断完善社会稳定风险评估、矛盾纠纷排查调处、群众利益诉求表达等群众工作机制，加强行政调处中心规范化建设，促进社会和谐稳定。抓好双拥共建和优抚安置工作，加强国防动员、民兵预备役、打击走私和人民防空建设。做好民族宗教、妇女儿童、外事、侨务、对台、档案、史志、地震、气象和对口支援等工作。

（六）深化行政体制改革。严格控制机构编制规模，健全部门职责体系，分类推进事业单位改革。深化行政审批制度改革，进一步清理和规范审批事项，优化行政审批和服务流程。大力推广电子政务，完善电子审批系统，增强网上办事功能。完善政务服务体系，在较大的村居推广建立便民服务代办点，推动便民服务向基层延伸。

七、大力保障和改善民生

坚持以人为本，按照“守住底线、突出重点、完善制度、引导舆论”的思路做好民生工作，解决好群众最关心、最直接、最现实的利益问题，在学有所教、劳有所得、病有所医、老有所养、住有所居上持续取得新进展，努力让人民群众过上更好生活。

（一）办好人民满意的教育。坚持教育优先发展，全面实施素质教育，着力提高教育质量。推动学前教育公益普惠发展，公办及公办性质幼儿园比例提高到60%，提高幼儿教师持证上岗率。实施学生素质提高工程，培育50所市级特色学校，培养学生创新精神。加强与驻威高校共建。促进威海职业学院内涵式发展。加快山东交通学院海运学院、威海海洋职业学院建设。支持民办教育规范发展。建立市平安校园防范监控中心，提升校园安全防范水平。实施“四名工程”，建立教师素质持续提高长效机制，切实提高师德水平和业务能力。

（二）推动实现更高质量的就业。实施就业优先战略和更加积极的就业政策，鼓励多渠道多形式就业，促进居民收入增长与经济发展同步。加强就业培训和就业援助，做好以高校毕业生为重点的青年就业工作和农村转移劳动力、城镇困难人员、退役军人就业工作，新增城镇就业3.3万人，转移农村劳动力1.5万人。健全劳动争议预警、调解和仲裁机制，依法保护劳动者合法权益，构建和谐劳动关系。

（三）提高人民群众健康水平。按照保基本、强基层、建机制要求，深化医药卫生体制改革，健全全民医保体系。完善新农合大病保险制度，提升基础保障能力。城镇居民医保大额医疗费用年度支付限额由10万元提高到17万元，大病报销比例达到70%，进一步减轻群众医疗负担。积极推进支付方式改革，推广家庭医生式服务模式，提高公共卫生服务质量。做好乳山市县级公立医院综合改革试点工作。开展“中医中药进万家”活动。加强医德医风建设，构建和谐医患关系。加强食品药品安全监管，提高食品抽样检测和风险检测覆盖率，开展餐饮服务食品安全和药品安全示范市建设。实施免费孕前优生健康检查项目，争取目标人群覆盖率达到80%以上，进一步提高出生人口素质。

（四）丰富人民群众精神文化生活。加强社会主义核心价值体系建设，做好青少年思想道德教育工作，全面提高公民道德素质。加强“诚信威海”建设，集中开展道德领域突出问题专项教育和治理，完善社会征信体系。深入开展全国文明城市创建活动，扎实推进乡村文明行动，广泛开展志愿服务，推动学雷锋活动、学习宣传道德模范常态化。推进基层公共文化服务体系建设，积极实施文化惠民工程，广泛开展群众性文化活动。完成“十艺节”承办和参赛任务，办好第七届国际人居节。全面完成农民体育健身工程，实现村村配备健身器材；建设市乒羽健身中心，广泛开展全民健身运动。举办长距离铁人三项世界杯赛、全国帆船冠军赛等体育赛事。加强文物保护工作，继续实施老洋房保护工程。

（五）统筹完善社会保障体系。健全社会保险关系转移接续制度，继续扩大各类社会保险覆盖面，稳步提高保障水平。完善城乡困难群众临时救助制度，支持发展慈善事业。继续提高企业退休人员基本养老金。大力发展养老事业，鼓励社会资本兴办养老产业，建设2处老年人福利中心，新建改造120处老年人日间照料中心。健全残疾人社会保障和服务体系，切实保障残疾人权益。实施福利化殡葬，免除764元的基本费用。认真执行国家房地产调控政策，加强保障性住房建设和管理，完善阶梯式住房保障体系，努力满足城镇中低收入家庭、新就业职工和进城务工人员的基本住房需求。

各位代表！人民对美好生活的向往，就是我们的奋斗目标；群众的需求，就是政府的工作方向。我们广泛征求社会各界的意见，顺应人民的期待，回应群众的呼声，今年集中力量办好12项民生实事：（1）利用三年时间，购置711辆节能环保型公交车，全部淘汰中心城区现有611辆“黄标车”、老旧公交车，其中今年购置230辆，中心城区智能化公交车比重达到40%以上；开通经区至高区大站快线等公交新线路3条、62.7公里，优化调整公交线路5条；开工建设公交停保场2座。（2）资助生活困难学生2万人；免收中等职业教育学校农村学生及涉农专业学生学费。（3）新建公办和公办性质幼儿园10所；建成并启用市中小学生综合实践基地；发展农村中小学新模式校车100辆以上。（4）城乡居民基础养老金标准由每人每月60元提高到70元。（5）提高困难群众保障标准，城市低保标准由每人每月400元提高到450元，农村低保标准由每人每年2400元提高到2600元；农村五保对象集中供养和分散供养标准分别提高到每人每年4500元和2700元。（6）新农合统一筹资标准由每人每年300元提高到350元以上。（7）建设农村标准化卫生室179处；中心城区新增社区卫生服务中心3个；完成市中医院外科综合楼扩建主体工程。（8）实施保障性安居工程，新建2000套公共租赁住房、180套经济适用房，新增廉租住房租赁补贴150户，实施棚户区改造1.4万户。（9）中心城区综合整治旧生活区16个，整治楼宇353栋、面积107.48万平方米；完成既有建筑供热计量及节能改造941.8万平方米；完成270个村的环境综合整治任务；集中建设农房1万户，补助改造农村危旧房2000户。（10）开工建设市图书馆新馆和群众艺术馆；完善300个农村文化大院服务功能；培训基层文化辅导员及文艺骨干3400余人；为全市农村和城市社区免费送戏500场次。（11）建设市级示范“放心粮店”56个，组建放心粮油配送中心3个，改造居民主食厨房3个，为农户提供储粮示范仓5000个。（12）新建改造农家店100处、社区便利店40处；启用市农副产品批发市场，推进农超、农批对接，受益农户新增1.2万户。这些民生实事是政府的公开承诺，我们将主动接受社会监督，接受群众评议，把实事办实、好事办好，努力通过政府的工作，一点一滴地改善群众生活，实实在在地提高群众的幸福感。

八、加强政府自身建设

各位代表！建设法治政府、服务型政府和廉洁政府，是政府对全市人民的庄严承诺。我们将一以贯之，把这一根本要求贯穿于政府工作的各方面和全过程，认真落实中央关于改进工作作风、密切联系群众的八项规定和省委、市委的实施办法，持续开展机关作风建设年活动，不断提高施政能力和服务水平，努力建设为民、务实、清廉的人民满意政府。

（一）坚持依法行政，深化法治政府建设。组织开展学习贯彻党的十八大精神活动，加强公务员队伍培训，增强学法尊法守法用法意识。坚持按程序议事、按制度办事，全面加强政府工作各领域、各环节的制度建设，切实规范政务运行。坚持科学民主依法决策，落实重大行政决策程序规定，健全规范性文件公示制度，扩大公众对政府决策的有效参与。完善行政执法程序，切实做到严格规范公正文明执法。推进相对集中行政复议权工作，支持群众通过合法途径理性表达诉求，切实保障人民群众权益。

（二）坚持高效施政，深化服务型政府建设。坚持以公众需求为导向，加快转变政府职能，使行政服务更加贴近民心、符合民意。政府工作人员要增强群众观念，增进群众感情，利用市长公开电话、市长信箱、行风热线、社情民意调查等各种形式主动倾听群众呼声，根据群众需求开展工作。建立群众监督员制度，聘请基层群众及时反映社情民意，帮助政府改进工作。着力整治慵懒散奢等不良风气，切实解决门难进、脸难看、事难办等问题。坚持服务下沉，完善服务承诺和限时办结制度，提高工作效率和办事透明度，让企业和群众不托人也能好办事、快办事、办成事。健全督查督办制度，完善绩效考核体系，以实打实的工作为群众谋利益。

（三）坚持从严治政，深化廉洁政府建设。全面落实党风廉政建设责任制，按照“一岗双责”要求，加强对干部队伍的教育、管理和监督。自觉接受人大的法律监督、政协的民主监督和群众的社会监督，认真办理人大代表建议和政协委员提案。加大政务公开力度，保障人民群众的知情权、参与权和监督权。健全岗位廉政风险防控管理机制，加强对政府投资项目、公共资源交易、财政资金使用等方面的跟踪审计。力戒奢靡之风，反对大手大脚、铺张浪费，从严控制“三公”经费支出规模和比例。严肃查处各类违纪违法案件，坚决纠正损害群众利益的不正之风，以优良的政风凝聚民心，赢得人民群众的信任和支持。

各位代表！绘就科学发展的蓝图，托起人民群众的梦想，是政府的使命所系、职责所在。让我们紧密团结在以习近平同志为总书记的党中央周围，在省委、省政府和市委的坚强领导下，脚踏实地，真抓实干，不断开创科学发展新局面，为加快建设现代化幸福威海而努力奋斗！
